# Improving classification of low-resource COVID-19 literature by using Named Entity Recognition

**DOI:** 10.5808/gi.21018

**Published:** 2021-09-30

**Authors:** Oscar Lithgow-Serrano, Joseph Cornelius, Vani Kanjirangat, Carlos-Francisco Méndez-Cruz, Fabio Rinaldi

**Affiliations:** 1 Dalle Molle Institute for Artificial Intelligence Research, IDSIA USI-SUPSI, Polo universitario Lugano-Campus Est, Via la Santa 1, CH-6962 Lugano, Switzerland; 2 Centro de Ciencias Genómicas, Universidad Nacional Autónoma de México, Avenida Universidad s/n Col. Chamilpa, 62210 Cuernavaca, Mor., Mexico

**Keywords:** classification, COVID-19, Named Entity Recognition, NLP

## Abstract

Automatic document classification for highly interrelated classes is a demanding task that becomes more challenging when there is little labeled data for training. Such is the case of the coronavirus disease 2019 (COVID-19) clinical repository—a repository of classified and translated academic articles related to COVID-19 and relevant to the clinical practice—where a 3-way classification scheme is being applied to COVID-19 literature. During the 7th Biomedical Linked Annotation Hackathon (BLAH7) hackathon, we performed experiments to explore the use of named-entity-recognition (NER) to improve the classification. We processed the literature with OntoGene’s Biomedical Entity Recogniser (OGER) and used the resulting identified Named Entities (NE) and their links to major biological databases as extra input features for the classifier. We compared the results with a baseline model without the OGER extracted features. In these proof-of-concept experiments, we observed a clear gain on COVID-19 literature classification. In particular, NE’s origin was useful to classify document types and NE’s type for clinical specialties. Due to the limitations of the small dataset, we can only conclude that our results suggests that NER would benefit this classification task. In order to accurately estimate this benefit, further experiments with a larger dataset would be needed.

## Introduction

The current pandemic took the world by surprise in all aspects, among which is the efficient broadcasting of relevant findings to reach interested researchers, clinicians and other stakeholders. The medical literature relevant to coronavirus disease 2019 (COVID-19) is growing exponentially and, doctors, clinicians, and health workers in general need tools for monitoring and prioritizing the literature to make the most of their time by allowing them to quickly identify the most relevant information.

We have witnessed an enormous effort and solidarity that experts around the world have put in contributing and sharing a broad scope of experiences and findings. In this paper we consider in particular the case of an interinstitutional COVID-19 cooperation group (https://covid19.ccg.unam.mx/) which has been working to provide tools and resources for COVID-19 literature exploration.

The main contribution of the group is a clinical repository consisting of continuously updated academic literature related to COVID-19 (https://covid19.ccg.unam.mx/repoinfo.html). Considering the PRECEPT [1] scheme, their clinical experience and, other common classifications, the group has adopted a 3-way classification strategy to organize the repository to make it easier for potential users to find relevant literature. Despite a big effort to read and manually classify these documents, the pace of production of this literature hamper the utility of this work. In response the group has been working on automatic document classification machine learning models (https://covid19.ccg.unam.mx/machinelearning.html), however the number of classes, the interconnected nature of the clinical fields and the very little labeled examples imposes considerable challenges [2,3].

On the other hand, another important contribution has been the inclusion of a specialized COVID-19 named-entity annotation service, OntoGene’s Biomedical Entity Recogniser (OGER) [4]. OGER is an annotation tool that performs Named Entity Recognition and Disambiguation (NERD) using shallow and deep dependency parsing combined with state-of-the-art machine learning techniques. It interacts with *The Bio Term Hub* (https://covid19.nlp.idsia.ch/bth.html) which enables OGER to process and link named entities from major life science databases: cellosaurus, cell ontology, ChEBI, CTD, EntrezGene, Gene Ontology, MeSH, Molecular Process Ontology, NCBI Taxonomy, Protein Ontology, RxNorm, Sequence Ontology, Swiss-Prot, Uberon.

Aiming to leverage the convergence of both contributions we hypothesized that the named entities recognized by OGER could be used as a free-lunch feature augmentation strategy to boost the classification performance [5,6].

During the Biomedical Linked Annotation Hackathon 7 (https://blah7.linkedannotation.org/) within the task “Analyzing COVID-19 literature with OGER” we proposed a subtask oriented to investigate this hypothesis (https://coree.github.io/blah7/task3.html).

The task’s aim was to classify COVID-19 literature according to three independent dimensions: clinical specialties, types, topics-and-subtopics, with special emphasis in exploring the use of Named Entities to better leverage the title, abstract and text during classification.

Here, we present a proof-of-concept experiment performed during the hackathon. We processed the literature with OGER and used the resulting identified named-entity-recognition (NER) and their links to major biological databases (NED) as extra input features for a basic classifier model. We then compared the results with the same classification model but without the OGER extracted features. Although very preliminary, the results are promising and show a clear gain on COVID-19 literature classification by using named-entity-recognition as an auxiliary feature augmentation step, suggesting the benefit of NER for this task.

## Methods

In the preprocessing phase the original PDF documents were converted to text using the Linux utility *pdftotext* and no other cleaning or normalization steps were performed.

Next, the documents’ title and full text were converted to features applying Term Frequency-Inverse Document Frequency (tf-idf), Latent Semantic Analisis (LSA), and using the CLS embedding (first embedding) of the pretrained BERT-base as a sentence-embedding [7].

Besides, the full-text was processed with the OGER’s API specialized for COVID-19 applications (https://pub.cl.uzh.ch/projects/ontogene/oger/upload/txt/tsv?dict=509f822aaf527390). This process resulted in an average of 1,622 annotations per article (The same token can be annotated as multiple entities because OGER uses multiple sources) including, among others, the entity type (e.g., organism, disease) (The full list of entity types annotated by OGER in this dataset were: organism, sequence, cellular_component, clinical_drug, cell_line, organ/tissue, gene/protein, chemical, cell, disease, molecular_process, biological_process, molecular_function), the matched term (e.g., coronavirus), the preferred-form (e.g., coronavirus), the entity ID and, the origin database (e.g., MeSH diseases, CTD MeSH). OGER has the capacity to return results in different formats, for our purpose TSV was the most convenient (Table 1 for an output example).

The classification task was approached as a multi-class problem for each of the three classification axis; this is, each document can be labeled with at most one topic, one type, and one specialty.

**Fig. 1.**
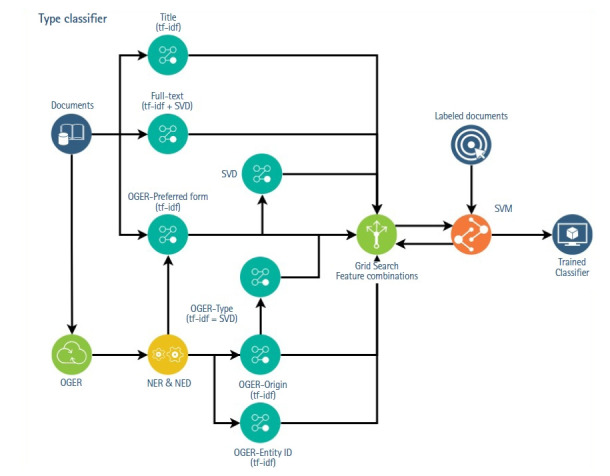
Schematic representation of the experiment pipeline. At the top half, feature extraction of documents, titles, and full-text was done with tf-idf and LSA techniques, respectively. At the bottom half, documents were first processed with OGER, and then different feature extraction strategies were applied over the extracted NERD fields. Tf-idf was applied to the origin and entity-ID values and LSA to preferred-form. Finally, an exhaustive search of feature combinations with an SVM classifier was performed. tf-idf, Term Frequency-Inverse Document Frequency; LSA, Latent Semantic Analisis; OGER, OntoGene’s Biomedical Entity Recogniser; NERD, Named Entity Recognition and Disambiguation; SVM, Support Vector Machine.

The general approach to use the NERD results consisted in treating each field of OGER output as an alternative representations of the documents and, then, apply different feature extraction over these representations (Fig. 1). To create each representation, all the values of one field of the OGER results were joined as words, with a space as separator. For example, to obtain the *preferred-form* representation of a document, all the value of the preferred-form field of all the identified entities in the document were concatenated. The resulting string is then treated as if it were an additional sentence in the input text and thus, converted using two basic feature extraction strategies: tf-idf and LSA.

The result was 11 features: six corresponding to the tf-idf, LSA and bert-embedding (Due to the BERT model limitation, we use only the first 512 tokens of the article body to build the text embedding) for the document’s title and the text representations, and 5 from the NERD extraction: tf-idf of the type, tf-idf and LSA [8,9] of the *preferred-form*, tf-idf of the *entity-id* and, tf-idf of the *origin*. These features were later reduced (independently for each classification axis) through a supervised exhaustive feature selection with a Support Vector Machine (SVM) [10] on a 3-fold cross validation, i.e., through a greedy search evaluating with the SVM classification model all possible combinations of features.

The final validation consisted in training the selected model (the SVM with the reduced features) on a stratified split corresponding to the 80% of the labeled dataset and, test it in the remaining 20%. This was repeated in 30 runs with random train-test split.

One possible limitation of this study is that we performed the search for the best features combination by 3-fold cross-validation in the full dataset. This was done because the dataset was very small, highly unbalanced and some classes had only three examples. Although in the final validation phase training and testing were done in clearly separated subsets of the original data, further experiments with more data are needed to validate the observed benefits.

### Dataset

The set of manually labeled documents from the clinical repository was used for these experiments.

It is worth noticing that the labeled dataset consists of 646 articles with a rounded average of 12 words for the title and 3,540 for the body. However not all documents are classified in each of the 3-classification axis: the *Document type* classification (e.g., observational studies, systematic reviews) has 269 examples for the 6 classes; the *Clinical specialties* classification (e.g., immunology, Cardiology) has also 269 examples but for 36 classes; and the *Topic & Subtopic* classification (e.g., epidemiology, diagnosis) counts 383 examples for the 16 topics and 161 examples for the 27 subtopics. Moreover within each classification axis the examples are not uniformly distributed through the classes.

## Results

The baseline was selected as the model with the best combination of features extracted from the document title and text, i.e., without the NERD results. In this case, the best baseline model was obtained by using the tf-idf of the title and LSA of the text.

For the Document type classification, the selected features were: tf-idf of the title, LSA of the text and tf-idf of the NE origin. The F1-weighted mean of the 30 validation runs was 0.739 (*s* = 0.041 [sample standard deviation]) and, by including the NE origin as feature the classifier had a significant 10% gain (t-test with p < 0.05) compared to the 0.669 (*s* = 0.044) F1-weighted score of the baseline (Table 2).

In the specialty classification the best model consisted of the features tf-idf of the title, LSA of the text and tf-idf of the NE type. This combination resulted in a statistically significant 8% gain in the F1-weighted mean compared to the baseline, 0.646 (*s* = 0.044) versus 0.597 (*s* = 0.048).

**Fig. 2.**
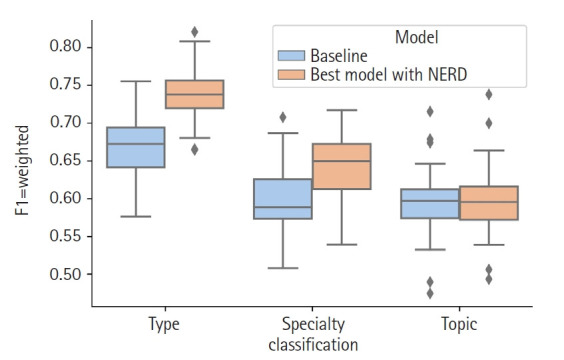
Comparison of results distributions between baseline and the best model using Named Entity Recognition and Disambiguation (NERD) features for each classification axis.

Finally, for the topic classification the best features were tf-idf of the title, LSA of the text and tf-idf of the NER origin. The F1-weighted mean of the 30 runs for this model was 0.599 (*s* = 0.051) whereas the baseline scored 0.595 (*s* = 0.049). Although this represented an improvement of 0.8%, it was not statistically significant. Fig. 2 shows the comparison of the results mentioned above.

## Discussion and Conclusion

It is worth noticing that the pdf to text conversion was done using a basic approach which produces quite noisy results, i.e., the extracted text includes lines that are not complete sentences due to how they are displayed in the PDF; it also includes statements interrupted by spurious chunks of text like footnotes, page numbers, etc. and other kinds of not cleanly extracted text. This might explain why BERT features played no role, and was detrimental to the OGER processing and the quality of its results.

Interestingly, we found that the named entities’ type and origin, i.e., more general features, were more informative for the classification than the more specific ones, like the matched term, the preferred-form, and the entity ID. One possible explanation is that due to the granularity of the general features, there are more examples in the training data and, thus, the estimator can better learn the relation between those features and the target classes. The fact that NE’s type (e.g., organism, disease) helped classify specialty may unveil an interesting pattern where some biomedical entities are more prevalent in some specialties. On the other hand, the fact that using the NE origin (MeSH diseases, CTD MeSH) improved document type classification is an interesting finding that should be further investigated. These experiments also opened the question of whether using BioBert Embeddings, trained for Biomedical Domain, instead of general Bert Embeddings may help classification.

It is important to stress that these were proof-of-concept experiments, and bearing in mind the methodological limitations due to the small dataset the conclusions here presented are only suggestive, and further experiments with more data are needed to accurately estimate the observed benefits of NER in the classification task.

Finally, it is important to highlight that due to the limited time in the hackathon, the experiments presented here were applied and compared to a baseline and not to the classification strategy that the COVID-19 cooperation group is developing. The next step would be to investigate if similar gains could be obtained when integrating NERD features in that strategy and applied to a larger dataset.

## Figures and Tables

**Table 1.** Fragment exemplifying an OGER output in TSV format

Type	Start	End	Matched term	Preferred form	Entity ID	Origin
Disease	42	50	COVID-19	COVID-19	C000657245	MeSH supp (Diseases)
Organism	126	137	Coronavirus	Coronavirus	D017934	MeSH desc (Organisms)
Disease	138	145	COVID19	COVID-19	C000657245	MeSH supp (Diseases)
Disease	151	159	COVID-19	COVID-19	C000657245	MeSH supp (Diseases)
Disease	309	317	COVID-19	COVID-19	C000657245	MeSH supp (Diseases)
Disease	360	368	COVID-19	COVID-19	C000657245	MeSH supp (Diseases)
Disease	733	741	COVID-19	COVID-19	C000657245	MeSH supp (Diseases)
Chemical	776	785	Antiviral	Antiviral agent	CHEBI:22587	ChEBI
Chemical	857	866	Antiviral	Antiviral agent	CHEBI:22587	ChEBI
Chemical	907	910	com	Coenzyme M	CHEBI:17905	ChEBI
Chemical	918	927	Antiviral	Antiviral agent	CHEBI:22587	ChEBI
Clinical_drug	944	955	Chloroquine	Chloroquine	2393	RxNorm
Chemical	944	955	Chloroquine	Chloroquine	D002738	CTD (MESH)

OGER, OntoGene’s Biomedical Entity Recogniser; COVID-19, coronavirus disease 2019.

**Table 2.** Best model results per classification axis

Classification axis	Baseline model	Model with NERD features
Document type	0.669 (*s* = 0.044)	0.739^a^ (*s* = 0.041)
Specialty	0.597 (*s* = 0.048)	0.646^a^ (*s* = 0.044)
Topic	0.595 (*s* = 0.049)	0.599 (*s* = 0.051)

Values are the average of the F1-weighted mean of 30 runs; s, sample standard deviation. NERD, Named Entity Recognition and Disambiguation.

a The difference is statistically significant.

## References

[1] Harder T, Sin MA, Bosch-Capblanch X, Coignard B, de Carvalho Gomes H, Duclos P (2015). Towards a framework for evaluating and grading evidence in public health. Health Policy.

[2] Prati RC, Batista GE, Monard MC. Class imbalances versus class overlapping: an analysis of a learning system behavior. In: MICAI 2004: Advances in Artificial Intelligence. MICAI 2004. Lecture Notes in Computer Science, Vol. 2972 (Monroy R, Arroyo-Figueroa G, Sucar LE, Sossa H, eds.). Berlin: Springer, 2004. pp. 312-321.

[3] Denil M, Trappenberg T, Farzindar A, Keselj V (2010). Advances in Artificial Intelligence. Canadian AI 2010. Lecture Notes in Computer Science, Vol. 6085.

[4] Basaldella M, Furrer L, Tasso C, Rinaldi F (2017). Entity recognition in the biomedical domain using a hybrid approach. J Biomed Semantics.

[5] Armour Q (2005). The role of named entities in text classification. M.A.Sc. Thesis.

[6] Andelic S, Kondic M, Peric I, Jocic M, Kovacevic A Text classification based on named entities.

[7] Devlin J, Chang MW, Lee K, Toutanova K (2019). BERT: pre-training of deep bidirectional transformers for language understanding.

[8] Deerwester S, Dumais ST, Furnas GW, Ladauer TK, Harshman R (1990). Indexing by latent semantic analysis. J Am Soc Inf Sci.

[9] Landauer TK, Dumais ST (1997). A solution to Platos problem: the latent semantic analysis theory of acquisition, induction, and representation of knowledge. Psychol Rev.

[10] Cortes C, Vapnik V (1995). Support-vector networks. Mach Learn.

